# Element Contents in Three Commercially Important Edible Mollusks Harvested off the Southwestern Coast of Crimea (Black Sea) and Assessment of Human Health Risks from Their Consumption

**DOI:** 10.3390/foods10102313

**Published:** 2021-09-29

**Authors:** Sergey V. Kapranov, Nadezhda V. Karavantseva, Nikolay I. Bobko, Vitaliy I. Ryabushko, Larisa L. Kapranova

**Affiliations:** A.O. Kovalevsky Institute of Biology of the Southern Seas of RAS, 299011 Sevastopol, Russia; nvideokaravan12@gmail.com (N.V.K.); ni.bobko@yandex.ru (N.I.B.); rabushko2006@yandex.ru (V.I.R.); lar_sa1980@mail.ru (L.L.K.)

**Keywords:** mollusks, *Mytilus galloprovincialis*, *Rapana venosa*, *Crassostrea gigas*, trace elements, ICP-MS, cluster analysis, accumulation universality index, human health risk

## Abstract

Mollusks are a prospective food for the world’s growing population, but the contents of toxic and essential trace elements in them have not been studied comprehensively. In this work, using inductively coupled plasma mass spectrometry, the contents of 72 elements in soft tissues of the edible mollusks *Mytilus galloprovincialis*, *Rapana venosa*, and *Crassostrea gigas* from the coastal area of the southwestern Crimea were estimated and compared with the maximum permissible levels. Element accumulation similarities were observed in the two bivalve species. Cluster analysis applied to the non-normalized contents allowed finding an optimal number of non-overlapping element clusters: 1 group of macroelements, 1–2 groups of trace elements, and 1–2 groups of ultratrace elements. As an outcome of this analysis, the element accumulation universality index was introduced, which demonstrated the accumulation universality decrease in the order: mussel > sea snail > oyster. An original approach to estimating the mollusk consumption rate was proposed to assess human health risks. Two possible consumption scenarios were identified for Crimean residents. From the expected consumption of all species in both scenarios, there are no health risks, but they are not excluded, within the 95% probability, from high consumption of mussels and sea snails in the pessimistic scenario.

## 1. Introduction

From the United Nations report [1], the world population is projected to reach 9.8 billion in 2050, and the global food demand will therefore steadily grow in the coming decades. On a global scale, fish and seafood products are the third major source of dietary proteins for human nutrition [2], and they currently account for 17% of the global yield of animal meat [3,4]. They are also a rich source of polyunsaturated fatty acids, vitamins, and minerals [5], which are essential for human health. However, the state of the world’s wild marine fish stocks has been deteriorating since the 1970s, and in 2017, 93.6% of the total number of stocks were fished maximally sustainably or overfished [6] (p. 143). Moreover, the Mediterranean and Black Sea region had the highest percentage (62.5%) of stocks fished unsustainably [6] (p. 144). Mariculture is a weighty alternative to wild fisheries; it can provide sustainable production of seafood and contribute a considerable deal to global food security [6,7,8,9]. In the last few decades, it has been rapidly developing, and in 2018, its share in the total production of aquatic animals reached 46% [6] (p. 34). Bivalve mariculture, though accounting for only 16% of the farmed seafood production (as of 2018) [2], has a remarkable incentive to increase its pace of growth because it provides a relatively cheap and thus affordable food [3], which will be of particular importance for the growing population of developing countries.

Furthermore, bivalves play a significant role in clarification of marine environment and extraction of nutrients (“detrophication”) [10,11,12]. The latter effect occurs through the grazing of bivalves and their subsequent harvesting and/or biodeposition of nutrients in the form of (pseudo-)feces. The nutrient extraction by bivalves, mainly by mussels, is successfully utilized to improve water quality and mitigate eutrophication [13,14,15]. Risks of phytoplankton depletion due to the possible nutrient limitation [16,17,18,19] impel aquaculturists to set up near-shore mollusk farms in or close to the nutrient-enriched water bodies such as enclosed bays and estuaries.

However, the nutrient enrichment of water is typically accompanied by contamination with other hazardous pollutants, e.g., pesticides and heavy metals, which can be accumulated in considerable amounts in marine animals through the food chains [20,21,22]. Some of these pollutants are extremely toxic to mammals. Moreover, bivalves and gastropods are excellent trace element accumulators, thereby serving as bioindicators of heavy metal pollution [23,24,25,26]. Therefore, mollusk consumers’ safety should be permanently monitored and protected by taking special measures at different stages of mollusk treatment, from harvesting to consumption [5]. There exist different national and international regulations that set maximum permissible levels and tolerable daily intakes of marine biotoxins and microbiological and chemical contaminants in food, including edible mollusks.

At the same time, many trace elements are essential in physiological amounts for living organisms, and the essential element deficiency results in different developmental pathologies [27,28,29]. By the beginning of the 1970s, about 20 elements were recognized as essential; in 1972, their number increased to 24 [30], and in 1985, 30 elements were already accredited with having physiologically essential functions [31]. Six of them are bulk structural elements (H, C, N, O, P, S), five are macrominerals (Na, K, Mg, Ca, Cl), three are trace elements (Fe, Cu, Zn), and the other sixteen are ultratrace elements. By the beginning of the 2000s, the list of definitely essential ultratrace elements (Cr, Se, Mo) was supplemented with fifteen possibly essential ones [32,33]. In 1996, according to the classification of the World Health Organization (WHO) [34], there were six known essential trace elements (Cr, Cu, Zn, Se, Mo, I), five probably essential trace elements (B, Si, V, Mn, Ni), and seven potentially toxic trace elements with possibly essential functions (Li, F, Al, As, Cd, Sn, Pb). The continuously updated information on physiological functions and essentiality of trace and ultratrace elements suggests that future research can provide evidence of the greater number of essential inorganic nutrients [35].

Thus, in view of the current and future challenges, there is a need in quantifying as many trace elements in soft tissues of edible mollusks as possible. On the one hand, this is due to the need for a comprehensive assessment of human health risks from mollusk consumption as the risks from only three most hazardous heavy metals (Cd, Hg, Pb) are most commonly assessed. On the other hand, the information on the trace element contents is important in the perspective of discovering new essential elements and searching for their promising sources among food commodities. The method of inductively coupled plasma mass spectrometry (ICP-MS) is perfect for this purpose as it allows for determination of the full suite of elements from lithium to plutonium within one instrumental scan.

The focus of this study is the element contents in soft tissues of three commercially important edible mollusks from the Black Sea: the Mediterranean mussel *Mytilus galloprovincialis* (Lamarck, 1819), the veined rapa whelk *Rapana venosa* (Valenciennes, 1846), syn. *Rapana thomasiana* (Crosse, 1861), and the Pacific cupped oyster *Crassostrea gigas* (Thunberg, 1793). The Mediterranean mussel is indigenous to the Black Sea, and both the veined rapa whelk and the Pacific oyster are alien species, whose natural range is the Pacific coast of Northeast Asia. The mussel and the oyster are the most promising objects of bivalve mariculture in the Mediterranean and the Black Sea [36], and the gastropod *R. venosa* has been harvested as seafood in all countries of the Black Sea region, but mainly in Turkey [37,38,39], which annually produced and exported, on average, about 10 thousand tons of this sea snail in 2004–2014 [39].

Trace element contents in these mollusk species were studied in a large number of works, but even in the most comprehensive ones, in terms of the spectrum of elements covered, the number of elements under study was below 40. In this work, from the perspective of new essential trace element discoveries and for an exhaustive assessment of human health risks due to the mollusk consumption, we report the contents of 72 elements in soft tissues of the mussel, oyster, and sea snail collected in one time period and at the same site in the northwestern Black Sea.

The human’s daily intake of trace elements with shellfish depends on the shellfish consumption rate, which changes annually and varies not only in different countries, but also across a country. It is fairly obvious that people living on the seaside or close to the sea are more accustomed to consuming seafood than those who live far from the sea. However, the information about seafood (and in particular, mollusk) consumption is typically obtained on an ad hoc basis from special consumer surveys or by state authorities, and it is not always up-to-date, readily available, and relevant to consumers from specific country area. In the most extensive open database of the Food and Agriculture Organization (FAO) of the United Nations [40], the latest data date back to 2013. In the present work, we develop an original universal approach, based on the FAO dataset, to the estimation of the mollusk consumption rate at the finer levels of the territorial division than the countrywide one. As an example, we apply this approach to assessing the health risks for the residents of Crimea, the main consumers of mollusks from the Crimean coast.

The goals of this work are (i) to characterize and compare the three mollusk species under study in terms of their ability to accumulate elements, (ii) to develop a methodology of comprehensively assessing noncarcinogenic health risks from the mollusk consumption for people living in particular, and possibly small, country regions, and (iii) to showcase the implementation of this methodology by the example of Crimea.

## 2. Materials and Methods

### 2.1. Reference Search

Relevant literature references containing data on trace elements in the three mollusk species under study were searched using Scopus and Google search engines with the following keywords: “trace elements”|“heavy metals”, “*Mytilus galloprovincialis*”|“*Rapana venosa*”|“*Rapana thomasiana*”|“*Crassostrea gigas*”. For the data on *Mytilus galloprovincialis*, we picked out the publications mainly from the period of 2010–2021 and related to the Black Sea and the Eastern Mediterranean. For the other species, the search area was the entire Atlantic and the publication time was not restricted.

### 2.2. Sampling and Analytical Sample Preparation

**Figure 1:**
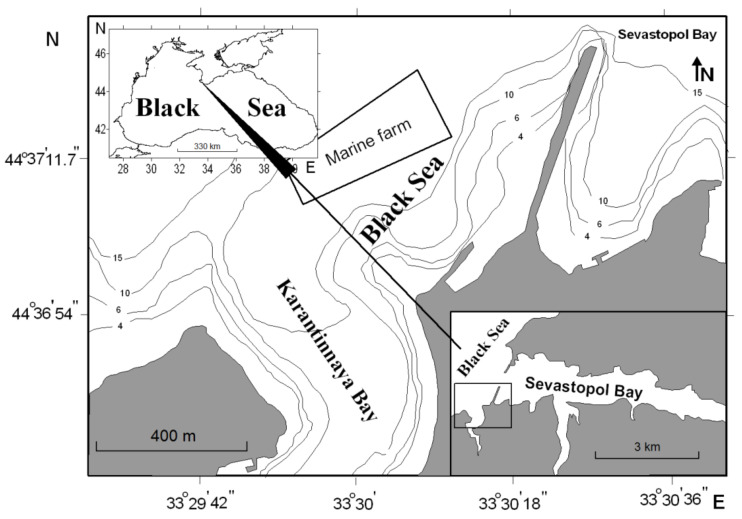
Mollusk sampling area in the Black Sea (marine farm). Lower right inset: position of the main panel (in rectangle). Upper left inset: sampling area location at the regional scale.

The mollusks of the commercial size were sampled on 3–5 May 2017, each species per day, from the area of the marine farm situated in the northwestern Black Sea off coasts of the southwestern Crimea (44°61′83.46′′ N, 33°50′33.80′′ E, Figure 1). The mollusk farm is situated between Sevastopol Bay and Karantinnaya Bay, which experience considerable anthropogenic loading [19]. The short 3-day sampling period was due to the requirement of sampling the mollusks from the environment with nearly constant physicochemical and ecological conditions. Late spring was chosen as the season of mass spawning of bivalves when the trace element contents in the mollusks are at their highest in the sexual ripening cycle [41].

The mussel specimens were harvested from the collectors suspended at a depth of 6 m, and the oyster specimens reared from spat were taken out of plastic cages hung at the same depth. The sea snails were collected from the bottom under the farm at a depth of about 18 m, where they fed on mussels fallen from the collectors.

The 2–3-year-old mussels (5 females and 5 males) with the shell length of 70–80 mm were taken at the pre-spawning stage 4 of the six-stage reproductive cycle [41,42]. Three diploid oysters with the length 10.58–13.46 mm were with empty gonads at stage 1 of the reproductive cycle, which did not allow us to identify their sex. Three sea snails (one male and two females) with the shell length of 7.92–8.81 mm and the approximate age of 5–8 years were at stages 2 and 3 of the five-stage reproductive cycle [43].

Sampled animals were transported to the laboratory in a plastic bucket with seawater within 1 h after the sampling and processed immediately after the transportation. Shells of the mollusks were cleared from incrustations and algae with a knife and wire brush in seawater. Bivalve shells were opened with a plastic scalpel, and shells of the sea snails were broken mechanically. The shell liquor was discarded. Soft tissues of the mollusks were dried in an oven at a temperature of 105 °C to the constant weight and homogenized in an agate mortar. Samples of dried soft tissues (~100 mg) were transferred into acid digestion tubes made of PTFE that had been preliminarily decontaminated by immersion in pure nitric acid (65%) and rinsed with deionized water (18.2 MΩ·cm). The samples were digested in 4.0 mL nitric acid (65%) of analytical grade additionally purified by sub-boiling distillation in an acid purification system DST-1000 (Savillex, Eden Prairie, MN, USA). The PTFE-capped acid digestion tubes were kept in an autoclave for about two hours. The digested samples were diluted with deionized water, with the final dilution factor being about 1000 mL·g^−1^ dry weight (d. w.).

### 2.3. Thermohaline and Hydrochemical Analyses of the Seawater Environment

The temperature and salinity of seawater in the sampling period were determined using a CTD probe “Katran-4” (Marine Hydrophysical Institute, Sevastopol) with an accuracy of at least ±0.01 °C in temperature and ±0.005‰ in salinity after the conversion of the measured quantities [44]. For the determination of hydrochemical parameters, seawater at the mollusk farm was sampled from a depth of 6 m using a sort of Nansen bottle, BM-48 (Meteopribor Ltd., Omsk, Russia), from which it was further dispensed in Winkler and BOD_5_ bottles and in polyethylene bottles for the oxidizability and nutrient determinations. The seawater samples were delivered to the laboratory within an hour after the sampling, and the hydrochemical properties were determined by means of conventional methods [45,46] immediately after the sample delivery (except for the five-day biochemical oxygen demand, BOD_5_).

A calibrated pH meter pH-410 (Akvilon, Podolsk, Russia) with an accuracy of 0.01 pH units was used for the pH measurements. The dissolved oxygen, BOD_5_, and alkaline permanganate oxidizability were determined by means of thiosulfate titration using a burette VITLAB^®^ continuous RS (Grossostheim, Germany) with an accuracy of 0.2%. The colorimetric determinations of nutrients were made on a spectrophotometer KFK-3-01 (ZOMZ, Sergiev Posad, Russia).

All reagents used in the analyses were of analytical grade. Potassium persulfate for the determination of organic nitrogen and phosphorus had been preliminarily triply recrystallized. Deionized water (18.2 MΩ cm) was utilized for dissolution and dilution. Certified standard solutions of nitrite, nitrate, ammonium, phosphate, and silicate ions (1 g L^−1^, *LenReaktiv*, St. Petersburg, Russia) were used for the calibration procedure.

### 2.4. ICP-MS Analysis of Elements and Statistical Analyses

The element concentration in the samples was determined using an ICP-MS instrument PlasmaQuant^®^ MS Elite (Analytik Jena, Germany). The detection limits in this method range from 0.2 ng·L^−1^ in solution and 0.1 ppb in tissues for some lanthanides (Pr, Tb, Tm, Lu) to 150–300 ng·L^−1^ in solution and 75–150 ppb in tissues for B and Se [47].

Certified multielement standards IV-ICPMS-71A–D (Inorganic Ventures, Christiansburg, VA, USA) were used to obtain the calibration curves. Mixed solutions of lithium and mercury (II) nitrates (Supelco, Bellefonte, PA, USA) were used as a separate standard. The coefficients of determination *R*^2^ of the linear regression curves for all elements were greater than 0.999.

The parameters of the ICP-MS instrument were the same as those indicated in Supplementary Information for [41]. We used the collision reaction interface (CRI) switched either off or on. In the “on” mode, the skimmer gas was hydrogen with a flow rate of 40 mL·min^−1^. CRI is used to eliminate most polyatomic interferences, and it improves the calibration curve linearity in case of such interferences, but decreases the signal and sensitivity. Among the analytes, 14 elements (B, Ca, V, Cr, Mn, Fe, Co, Br, Rb, I, Cs, La, Ce, Pb) were quantified with CRI turned on as the *R*^2^ coefficients of the calibration plots were higher while the signals of these elements were still strong enough in this mode.

In the analysis, no internal standard was used since the undesirable matrix effects were not expected at such a high dilution. To take into account the signal drift, the concentrations in the diluted standards IV-ICPMS-71A–D were controlled after each fifth sample and the interpolating polynomial relationship was used to correct the apparent concentrations in time.

Each biological object was analyzed in triplicate. Since the variances of the biological replications were much greater than those of the analytical ones, we used analytical means to find the total means and uncertainties at the 95% level.

The accuracy and precision of the method for the quantification of some elements were verified by the analysis of the certified European Reference Material ERM^®^-CE278k (tissue of mussel *M. edulis*, *n* = 5) from the Institute for Reference Materials and Measurements (Geel, Belgium). Samples of Reference Material (0.1 g) were digested in the extra pure nitric acid and diluted with deionized water according to the procedure described above. The certified and observed recoveries of the elements and the uncertainties at the 95% level are shown in Table 1.

All fits and prediction bands were obtained in GraphPad Prism 8.0.1. Statistical comparisons of fits were made using the extra sum-of-squares F test.

### 2.5. Multivariate Statistics: Cluster Analysis

To assess the grouping of elements according to their contents in soft tissues, we take advantage of cluster analysis. Two clustering methodologies are applied in this study: the agglomerative hierarchical and centroid-based clustering. These methods enable identification of families of elements whose contents are comparable due to geochemical abundance or similar bioaccumulation patterns.

To perform the agglomerative hierarchical clustering, the Matlab function *linkage* is used. Starting from individual objects as singleton clusters, the function iteratively merges every two most similar clusters in nodes until all nodes are linked in a single cluster (root). The graphical representation of the cluster linkages is the binarily-ramified tree, which is called a dendrogram. The node height in the dendrogram is a measure of dissimilarity of two linked clusters. In this work, the Euclidean distance between clusters is used as dissimilarity metrics and Ward’s method as a strategy of selecting two clusters to merge at each iteration step.

The centroid-based clustering, namely, the *k*-means algorithm used as the Matlab function *kmeans*, seeks to partition objects in *k* mutually exclusive clusters so that the objects within a cluster are as close to each other as possible. The position of each cluster’s centroid is adjusted by the algorithm to minimize the sum of distances from the centroids to the objects of the cluster. To ensure the global minimum of this sum, the function is run in 10,000 replications with the random initial position of each centroid.

The optimum number of the non-overlapping clusters is calculated in this work using logarithm of a sum of within-cluster sums of distances from centroids [45]:

(1) $$\Upsilon \left(k\right)=\ln\sum_{i=1}^{k}\sum_{x_{i}\in C_{i}}d\left(x_{i},c_{i}\right)$$

where $x_{i}$ and $c_{i}$ designate objects and centroid of a cluster $C_{i}$, respectively, and $d\left(x_{i},c_{i}\right)$ is the distance between the objects and the centroid. The optimum number of clusters is the nearest integer to the elbow of this function, which is found as the intersection point of two linear fits with the smallest sum of their mean squared residuals. Typically, the number *k* corresponding to this smallest sum is also equal to the function elbow integer. The examples of these plots are shown in Figure S1 in the Supplementary Materials.

### 2.6. Human Health Risk Assessment

The human health risk is commonly assessed using the rate of daily pollutant consumption per body weight unit—the estimated daily intake (EDI) [48,49].

It is calculated in mg pollutant·kg^−1^ human body weight·day^−1^ as

(2) $$\mathrm{EDI}=\mathrm{CR}\times C/{\mathrm{BW}}_{a}$$

where CR is the food consumption rate, i.e., the average weight of daily consumed food (in kg·day^−1^·capita^−1^), C is the pollutant content in the food (in mg·kg^−1^), and ${\mathrm{BW}}_{a}$ is the average human body weight assumed to be 70 kg.

The calculated EDI values should be compared with the reference data: provisional tolerable daily intake (PTDI) adopted by FAO/WHO [50,51,52], tolerable upper daily intake (UDI) introduced by European Food Safety Authority (EFSA) [53], or oral reference dose (RfD_o_) set by the United States Environmental Protection Agency (USEPA) [54]. If tolerable intake is given on the basis of another time period (week, month), then it can be reduced to daily intake by dividing by the corresponding number of days. The tolerable upper daily intake (UDI) is typically expressed in mg·day^−1^ and, for the comparison with EDI, should be divided by the body weight (70 kg).

The target hazard quotient (THQ) introduced by USEPA shows how much the estimated daily intake of a contaminant exceeds its upper oral reference dose:

(3) $$\mathrm{THQ}=\mathrm{EDI}/{\mathrm{RfD}}_{o}$$

The THQ values below 1 indicate no likely toxic effects of this contaminant in the long-term dietary exposure of a consumer.

The overall noncarcinogenic risks from the consumption of multiple contaminants are assessed using a hazard index (HI), which is a sum of the THQ values for each contaminant [55]:

(4) $$\mathrm{HI}=\sum_{i}{\mathrm{THQ}}_{i}$$

Again, the values of HI < 1 signal no likely intoxication risks to human health from the prolonged consumption of the contaminated food.

## 3. Results

### 3.1. Thermohaline and Hydrochemical Conditions at the Sampling Site

The mean thermohaline and hydrochemical characteristics of seawater at the sampling site in April–May 2017 found using standard methods [42,43] are presented in Table 2. All characteristics are typical for this season and are close to the long-term seasonal means [19]. The ratio of the mineral nutrients N:Si:P ≅ 20:18:1 indicates the phosphorus and silicon limitation in the phytoplankton development due to the starting phytoplankton bloom in this period, which is also manifested in the high oxygen saturation (>100%) owing to the active photosynthesis. Relatively high values of the oxidizability versus lower BOD_5_, too, indicate the prevalence of difficult-to-oxidize organics (phytoplankton cells) in the total organic matter. Thus, bivalves, and consequently, *R. venosa* predating on them, are abundantly provided with food and thrive. The temperature value demonstrates that bivalves are close to spawning or have just spawned.

### 3.2. Element Contents in Soft Tissues of the Mollusks

The element contents in pooled soft tissues of the mollusks are shown in Table 3. For comparison, we add several reference data. These are the maximum permissible values adopted in the Russian Sanitary Rules and Regulations (SanPiN, Cu: 30.0, Zn: 200.0, As: 2.0, Cd: 2.0, Hg: 0.2, and Pb: 10.0 mg·kg^−1^ w.w.) [56] and in the European Commission Regulation [57], contamination-indicative contents (CIC) of heavy metals in mollusks [58], and action levels established by the United States Food and Drug Administration [59]. The maximum permissible levels were transformed from the wet weight basis to the dry weight basis using the conversion factors: 6.29 for *M. galloprovincialis* [60], 4.18 for *R. venosa* [61], and 9.47 for *C. gigas* [62].

It is seen that in the dried soft tissues of the mussel *M. galloprovincialis*, the contents of Cr, Ni, Zn, As, Cd, and Hg are above CIC, but none of the element contents exceeds the maximum permissible levels from the Russian and European regulations. In dry soft tissues of the oyster *C. gigas*, there are four elements with the contents exceeding CIC (Cu, Zn, Ag, and Hg), and moreover, the contents of Cu and Zn are, respectively, 4.0 and 2.6 times higher than the maximum permissible levels established in the Russian regulations. In the sea snail *R. venosa*, of major concern are Cd, Hg, and Pb, which are above the corresponding CIC, with the Cd content exceeding the maximum permissible values from both the Russian and European regulations and Hg being above the Russian maximum permissible value. These results suggest that the sea area in question is likely contaminated with such heavy metals as Zn, Cd and Hg, two of which are extremely toxic for humans. Additionally alarming is the very high content of copper and silver in the cultivated oysters.

Additionally, the metal pollution index (MPI) was calculated as follows [63]:

(5) $$\mathrm{MPI}={\left(\prod_{i=1}^{n}M_{i}\right)}^{1/n}$$

where $M_{i}$ is the content of *i*th metal in the tissues (in mg·kg^−1^). MPI > 1 indicates the overall metal pollution. The list of metals used in this calculation was limited by those mentioned as hazardous in [54] (i.e., to which RfD_o_ values are assigned): Li, Be, Al, V, Mn, Fe, Co, Ni, Cu, Zn, Sr, Zr, Mo, Ag, Cd, Sn, Sb, Ba, La, W, Hg, Tl, Pb, U. The metal pollution index turns out to be above 1 for all the three mollusks (Table 1), which means the overall pollution with these metals.

**Figure 2:**
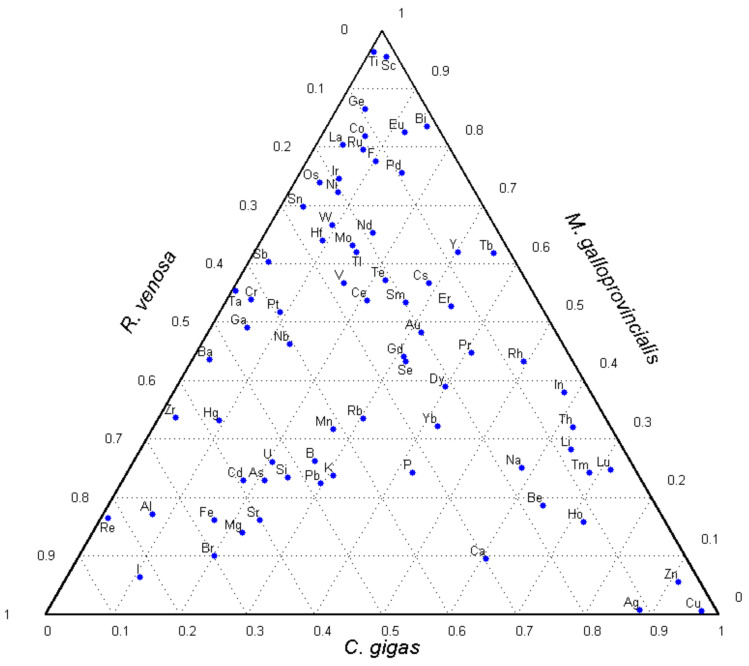
A ternary diagram of the element content fractions in soft tissues of the three mollusks.

To visualize the element accumulation patterns in the three mollusks, a ternary diagram is plotted (Figure 2), with the dots denoting the fractions of the element contents in the sum. To find these fractions for each mollusk, one should draw lines through the dot centers at angles 0°, 120°, and 240° to the horizontal. These lines will cut off, on the corresponding sides, the element content shares in soft tissues of the mussel, sea snail, and oyster, respectively. For example, the proportions of the Ca content are 0.1 for *M. galloprovincialis*, 0.3 for *R. venosa*, and 0.6 for *C. gigas*, together making up exactly 1.

From the diagram, it is seen that the mussel is the most efficient accumulator of Ti and Sc and the oyster is superior among the three mollusks in accumulating Cu, Zn, and Ag. The sea snail contains the highest relative contents of Re and I. A bunch of dots corresponding to various elements near the upper apex of the triangle signifies their high fractions in the mussel and means that *M. galloprovincialis* is the most universal element accumulator among the three mollusk species. The fraction value at which the largest share of each element is assigned to only one mollusk is 0.424. At this fraction, the numbers of elements corresponding to *M. galloprovincialis*, *R. venosa*, and *C. gigas* are 39, 20, and 13, respectively, which rate the overall element accumulating ability of the mussel to be two and three times higher than those of the sea snail and the oyster, respectively.

Bisectors in this triangle are lines in which the relative element contents in two mollusk species are equal. It is seen that a group of elements are close to the left angle bisector, which corresponds to the equal element fractions in the mussel and oyster. A large number of these elements are essential or probably essential, e.g., Mg, K, Mn, Fe, Si, As, Se, I, Br. Some other elements are chemically close to the essential ones (Sr and Mg, Rb and K) and can mimic them in tissues. This gives strong evidence in favor of a hypothesis that many essential elements are needed in equal proportions and play similar physiological roles in soft tissues of different bivalve species. In addition, there are a number of toxic heavy metals (Cd, Pb, U) close to this bisector, which indicates their similar accumulation pathways and content restrictions in the bivalves.

### 3.3. Cluster Analysis of the Element Contents and the Accumulation Universality Index

**Figure 3:**
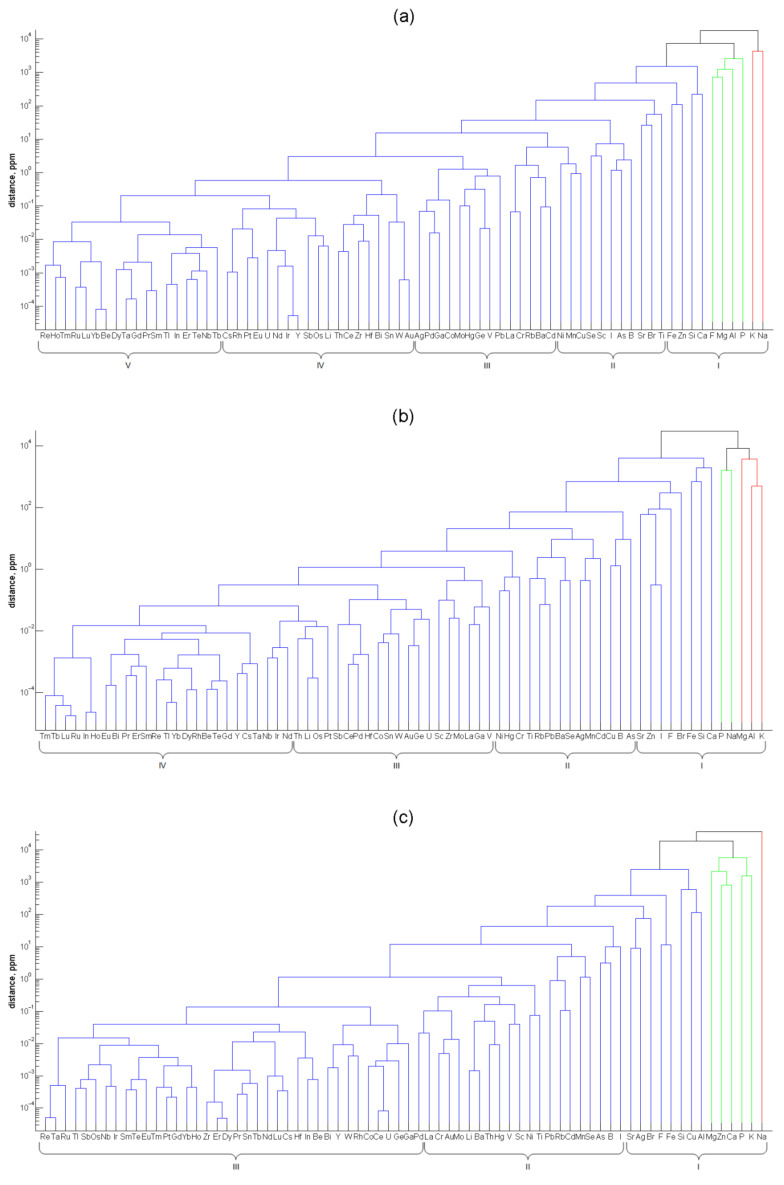
Dendrograms of the non-normalized element contents in soft tissues of (**a**) *M. galloprovincialis*, (**b**) *R. venosa*, and (**c**) *C. gigas*. The partition in the optimal number of clusters denoted by Roman numerals is shown below each panel.

Agglomerative hierarchical analysis using Ward’s linkage was applied to non-normalized element contents in soft tissues of the three mollusks. The dendrograms for the three species (Figure 3) are unilateral due to the steady decrease of the element abundance, from macrominerals to ultratrace elements, which mainly consist of refractory, noble and rare earth metals. The order of elements in the dendrograms of the three mollusks differs. For example, the titanium content in the mussel is high and approaches that of iron. In the sea snail and, especially, in the oyster, the titanium content is much lower and is close to the levels of such non-abundant trace elements as rubidium and lead. Another example of the elements with highly different levels in the mollusks’ soft tissues is silver. In the mussel tissues, its content is as low as that of the precious metals Au and Pd. In the sea snail, it is higher and approaches the content of the essential trace element Mn, and in the oyster, it is extremely high, being of the same order of magnitude as the strontium content. In general, the greatest differences in the element abundances in the mollusks are found for those elements which are close to the apices of the ternary diagram in Figure 2.

Additionally, the *k*-means clustering of the elements was performed. The number *k* of mutually exclusive clusters was changing from 1 to 11, and the sum of distances to the centroids was calculated each time. The optimum number of clusters was obtained as the integer nearest to the elbow in the logarithm of this sum plotted against *k* [45], as shown in Figure S1 in the Supplementary Materials. The partition of the elements in the non-overlapping clusters is given below each panel in Figure 3. The optimal number of non-overlapping clusters decreases from 5 for the mussel through 4 for the sea snail to 3 for the oyster. This order reflects the decreasing number of elements with the largest shares among the mollusks in this sequence. Indeed, the efficient element accumulation results in the bunching of elements in finer groups with comparable contents.

The elements included in cluster I can be referred to as macrominerals, and the lower boundary of the element contents in this cluster is in the range of 51(Sr, oyster)–197(Fe, mussel) ppm. Trace elements make up two clusters (II and III) in the mussel and one cluster (II) in the sea snail and oyster. The range of the element abundances in the mussel clusters III and II are 0.48(Ag)–3.88(Cd) and 5.2(Ni)–96(Ti) ppm, respectively. The element content ranges in cluster II of the sea snail and oyster are 1.47(Ni)–37(As) and 0.13(La)–24(I) ppm, respectively. The elements in the mussel clusters IV and V, sea snail clusters III and IV, and oyster cluster III can be denominated ultratrace elements. Their upper levels are 0.28(Au, mussel), 0.55(V, sea snail), and 0.11(Pd, oyster) ppm. In fact, this classification of elements according to their position in the non-overlapping clusters is close to the generally accepted ranking: from the definition of the International Union of Pure and Applied Chemistry, trace elements are those with the concentration less than 100 ppm (μg·g^−1^) [64], and ultratrace elements are typically defined as those with the mass fraction below 1 ppm [65], although there exists no strict definition for this group.

**Figure 4:**
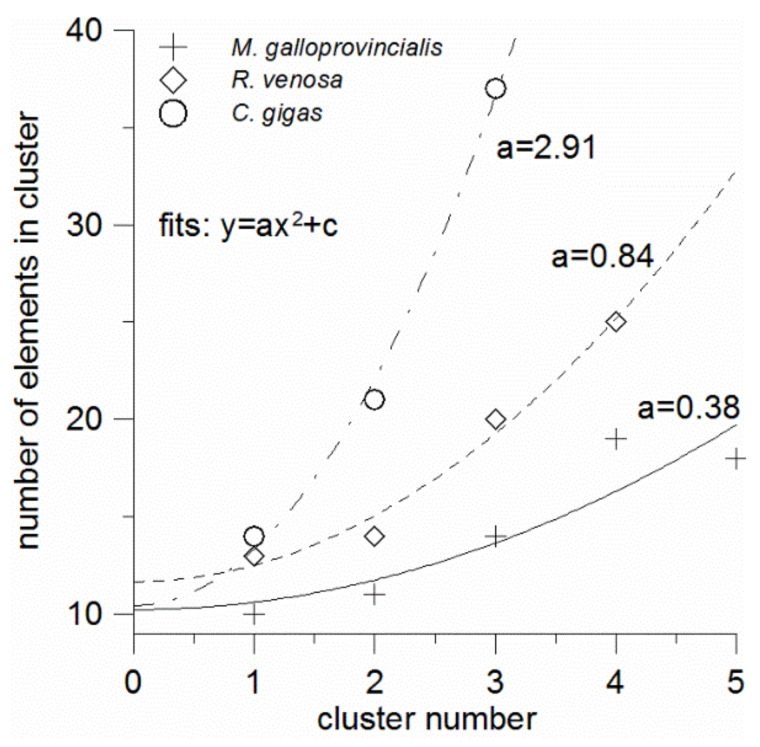
Number of elements in optimal non-overlapping clusters against the cluster number for the three mollusk species (legend). Lines: quadratic fits y = ax^2^ + c. The quadratic term coefficients are shown beside each fit curve.

The number of elements in clusters tends to increase with the cluster number, i.e., with the element abundance decrease. When plotted against the cluster number, the number of elements is seen to be well fitted with a parabola of the form y = ax^2^ + c for all the mollusks (Figure 4). It is seen also that the constant terms (c) of these equations are relatively invariable and equal to 10–12. This term has the meaning of the number of elements in the “zero” cluster, which seems to contain the unquantified bulk elements (C, O, N, H, S) possibly combined with some macrominerals. In contrast, the quadratic term coefficients (a) strongly depend on the mollusk species that formed the element clustering. They increase in the order: mussel (a = 0.38)—sea snail (a = 0.84)—oyster (a = 2.91); thus, from the mussel to the oyster this coefficient increases almost eight-fold. It is clear that it depends on how rapidly or slowly the numbers of elements increase in the successive clusters, which trend is related to the number of the optimal non-overlapping clusters. As mentioned above, the number of the non-overlapping clusters can be considered as the measure of the element accumulation universality, by which we mean the overall ability of a species to accumulate as many elements in its tissues as possible in comparison with other species. And the less universal accumulator has the greater coefficient a.

**Figure 5:**
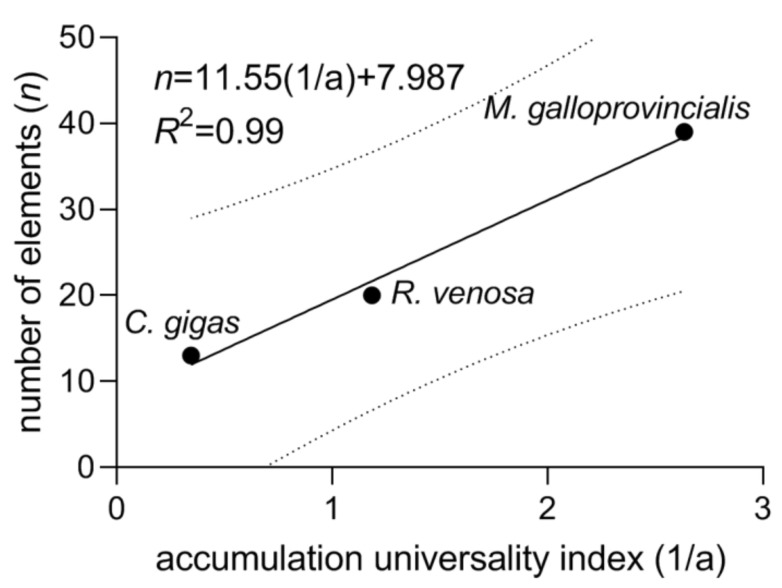
Number of elements with the largest shares among the three mollusks correlated with the element accumulation universality index. Solid line: linear fit; dotted lines: 90% prediction band boundaries.

Thus, the quantity 1/a can be introduced as the element accumulation universality index (Table 3). The reciprocal (a), in its turn, can be termed the element accumulation selectivity index. The accumulation universality index is expected to be positively correlated with the numbers of elements that are in largest proportions among the three mollusks (Section 3.2), and the linear correlation is indeed observed (Figure 5). From Figure 5, in the least universal accumulator (or ideal selector) with 1/a = 0, the number of elements whose shares are largest among any three mollusks from the same geographic area is with the 90% probability not greater than 27 (as it follows from the extrapolation of the upper prediction band boundary to zero), and the expected value is 8. Of course, the numbers of elements and the fit equation can be different in other environmental conditions, with a different set of elements and number of species under study, but the correlation linearity is expected to persist.

### 3.4. Estimation of the Mollusk Consumption Rate

As a starting point, we use the averaged mollusk consumption rates in 28 Eurasian countries with nonzero coastline length in the five-year period of 2009–2013 extracted from the FAOSTAT database [40]. Particular attention is paid to the countries of the Mediterranean and Black Sea regions. It can be hypothesized that the mollusk consumption rate per capita is positively correlated with such readily available quantities as the average salary approximated by gross domestic product per capita at purchasing power parity (GDP), coastline length (L), and population density (PD). The GDP and PD values for 2009–2013 are available from the World Bank Data [66], and the coastline length is given in [67]. The GDP role in the consumption rate is fairly straightforward: the greater the incomes, the greater sums of money are spent on food, including seafood. The expected correlation with the coastline length is due to the greater number of marine farms that can be established along a longer coastline. The expected correlation with the population density is explained as follows. Bivalve farming is one of the least costly options in mariculture [3] and it is most important for developing countries with the most rapidly growing population. However, people living closer to the seaside are more accustomed to consuming sea mollusks, and it is more correct to correlate the consumption rate with population per unit distance inland from the coastline. Upon division by the second direction, which is assumed constant, this linear population density is transformed into the regular surface density.

**Figure 6:**
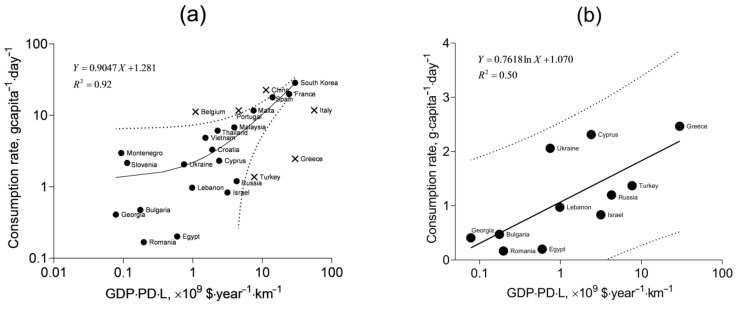
Mollusk consumption rates correlated with the gross domestic product per capita at purchasing power parity (GDP), population density (PD), and coastline length (L): (**a**) all selected countries, linear fit, and (**b**) low-consuming countries, logarithmic fit. Dotted lines: 95% prediction band boundaries. Crosses: outliers neglected in the fitting.

In Figure 6a, the consumption rates of the countries are shown against the product GDP·PD·L (Table S1, Supplementary Materials). It is seen that the linear function fits well most of the data, but some points remain beyond the 95% prediction band, and they are not included in the fitting. One group of the outliers corresponds to the countries with traditionally high mollusk consumption: Belgium, Portugal, and China. Another group is represented by the countries with low consumption rates and large values of GDP·PD·L: Turkey, Greece, and Italy. At the same time, the data for low-consuming countries, including Turkey and Greece, appear to follow a specific pattern different from that represented by the linear correlation in Figure 6a. This pattern is shown in Figure 6b. Statistical comparison of the linear fit from Figure 6a with one for the data in Figure 6b (Table S2, Supplementary Materials) shows their significant difference (*p* < 0.0001, *F* = 45.07). The low-consuming countries are washed by the waters of the Black Sea and the Eastern Mediterranean. It is likely the particular qualities of climate, mariculture and dietary traditions that determine the lower mollusk consumption rates in these regions. The correlation with the product GDP·PD·L for these countries is best described with the logarithmic fit rather than with the linear one (*R*^2^ = 0.50 and *R*^2^ = 0.45, respectively).

The higher consumption rates modeled by the fit in Figure 6a entail the higher intake of toxic elements, and this situation will be referred to as the pessimistic scenario, whereas the lower consumption rates modeled by the fit in Figure 6b will represent the optimistic scenario.

From the fits and the 95% prediction bands shown in Figure 6, it is possible to estimate the minimum, maximum, and mean (expected) consumption rates, given the GDP, PD, and L for specific territories. For Crimea, GDP is meant as the average annual salary (converted to US dollars) in the region. At the time of the mollusk sampling (2017), it was USD 5386 [68,69]. The population density (PD) in Crimea was 87.15 people·km^−2^ in 2017 [70], and the coastline length of the peninsula in the Black Sea is 945 km [71]. Thus, the product of these quantities is 0.44 × 10^9^ USD·year^−1^·km^−1^, and it corresponds to the mean of 1.7 g·capita^−1^·day^−1^ according to the pessimistic scenario and 0.8 g·capita^−1^·day^−1^ according to the optimistic scenario. The respective maximum values within the 95% prediction band are 6.8 and 2.3 g·capita^−1^·day^−1^. The corresponding minimum values are 0 g·capita^−1^·day^−1^.

### 3.5. Assessment of Human Health Risks from the Mollusk Consumption

The long-term health risks for the Crimean population from mollusk consumption can be assessed by means of Equations (2)–(4). In the calculation of the estimated daily intake (EDI), we use the expected and maximum values of the mollusk consumption rate according to both the pessimistic (EDI_1_) and optimistic (EDI_2_) scenarios. The minimum EDI values in both scenarios are 0 mg·kg^−1^_body weight_·day^−1^. The found consumption rate is applicable to the consumption of all mollusks since many people are conservative in their food preferences and therefore selective in consuming a certain mollusk species. Because food is commonly understood as a product ready for partaking, EDI should be calculated using the element content in the cooked form if cooking is relevant. In the cooked meat of the mussel and the sea snail, the factors of conversion from dry to wet weight are 3.45 and 3.82, respectively [60,61]. Oysters are typically consumed in raw form and the corresponding conversion factor is 9.47 [62]. The resulting EDI values are shown in Table S3 of the Supplementary Materials.

In Table S3, the calculated EDI values are juxtaposed with the tolerable intakes adopted by the US Environmental Protection Agency [54], World Health Organization [50,51,52,72,73], and European Food Safety Authority [53,73,74,75,76,77]. In the EDI for As, only the toxic inorganic arsenic was taken into account by multiplying the total arsenic content by 0.09 for the mussel and the oyster and by 0.07 for the sea snail [78]. From Table S3, it is seen that it is only maximum EDI_1_ for La in the mussel’s soft tissues that exceeds the corresponding RfD_o_. Hence, it is not unlikely that the long-term exposure, under the pessimistic consumption scenario, can lead to the chronic lanthanum intoxication.

The resulting target hazard quotients (THQ) calculated from Equation (3) and the hazard index (HI) from Equation (4) are presented in Table 4. The largest contributions to HI (in the decreasing order) are made by La, F, As, Cd in the mussel; I, Al, Cd, La, As, Hg in the sea snail; and Cu, Zn, Ag in the oyster. All expected values of HI in both the optimistic and pessimistic scenarios are below 1, and only two maximum values in the pessimistic scenario (for the mussel and the sea snail) are above 1. Thus, it is likely that the mollusk consumption cannot pose risk to health of the Crimean residents, but in the pessimistic scenario, this health risk from the mussel and sea snail consumption cannot be ruled out with the 95% probability.

## 4. Discussion

Trace elements in soft tissues of the Mediterranean mussel from the Mediterranean and Black Sea regions were studied in a large number of works, no fewer than thirty in the past decade. We will focus only on reports dealing with the mussels from the Black Sea and the Eastern Mediterranean in order to interweave the present local data with the regional perspective. The results of these studies are summarized in Table 5. The contents of several elements in the present work (Cr, Ni, Cu, As) are comparable with the results of other authors, whereas the contents of other elements are distinctly higher (Al, Zn, Ag, Cd, Hg) or lower (V, Mn, Fe, Co, Pb) than the respective medians over the region.

In Table 6 and Table 7, we present the element contents in soft tissues of the veined rapa whelk and the Pacific cupped oyster, respectively. The publications on the element abundances in these two mollusks, especially in the oyster from seas of the Atlantic basin, are not as numerous as the reports concerning the Mediterranean mussel. For this reason, we picked out the studies of wider spatial and/or temporal span: it is the Black and Marmara seas and the period of 1997–2017 for *R. venosa*, and it is the entire Atlantic basin and the period since the early 1970s until 2017 for *C.gigas*. It is seen that in the sea snail, above the medians are Cr, Fe, Zn, As, Cd, Hg, Pb and below the median is only Co. In the oyster, above the medians are Al, Zn, Cd, Hg and below the medians are Cr, Mn, Fe, Ni, As. Thus, the higher contents of Zn, Cd, and Hg in the three mollusks confirm the above-mentioned evidence (Section 3.2) of the water contamination with these metals. A noteworthy feature of the Pacific cupped oyster is its ability to accumulate quite large amounts of silver, which was also manifested by a close relative of this species, *C. virginica* [79]. Record-high levels of copper and zinc among the three mollusks are also typical for the oyster regardless of the region.

The element patterns, i.e., the groups of elements with the levels above or below the medians, in the mussel and oyster are similar, which indicate similar accumulation pathways in these bivalves. The lower contents of regular steel alloy components such as Fe, Mn, Co, Cr may be a consequence of the lack of mining industry and low shipyard and seaport activities in this area. This may also be a sign that the increased levels of other elements in these mollusks are probably due to natural reasons, rather than to active anthropogenic pollution.

In contrast to the bivalves, in the gastropod *R. venosa*, a larger number of elements demonstrate higher levels than the medians. This may be not only due to bioaccumulation through the predation on bivalves, but also because of increased element intake by this benthic mollusk from sediments. For example, larger contents of Cr, Fe, and Pb, which are not noted in the other two mollusks, may indicate higher levels of these metals in the sediments than those typically occurring.

**Table 5:** Element contents (min–max) in soft tissues of the mussel *Mytilus galloprovincialis* collected in the 2010s in different areas of the Eastern Mediterranean and Black Sea. NW = northwestern, NE = northeastern, SE = southeastern, etc. BDL: below detection level. Bold (red shading)/italic (blue shading) emphasis: mean values distinctly higher/lower than the respective medians over the region.

Sea (Area)	Year	Reference	Al	V	Cr	Mn	Fe	Co	Ni	Cu	Zn	As	Se	Sr	Ag	Cd	Sn	Sb	Ba	Hg	Pb
NW Black (Crimea, Russia)	2017	This work	**90–23,000**	*0.37–1.94*	1.67–5.45	*0.91–13.7*	*48–305*	*0.03–1.13*	0.38–9.0	0.67–14.9	**12.5–636**	6.7–21.8	4.3–17.5	10–132	**BDL–0.95**	**BDL–7.7**	0.09–0.7	BDL–0.22	0.5–15	**0.06–2.2**	*0.16–4.8*
NE Black (Kuban, Russia)	?	[80]	210–23,000	2.3–3.4	0.32–0.48	2.09–6.01	57–206	0.47–0.69	1.14–1.83	4.5–4.7	106–196	1.86–3.3		13.5–17.5		1.9–3.4			2.9–5.0	<0.05	0.39–0.53
SE Black (Giresun, Turkey) ^a^	2011	[81]			2.8–6.1	36–68	584–2224	13–18	56–160	16–26	396–650	20–27	2.4–8.1								20–29
S Black (Sinop, Turkey)	2009–2010	[82]								0.33–1.83	9.4–182.6					0.09–3.64					0.002–3.872
S Black (Sinop, Turkey)	2010	[83]								2.4–4.8	79–163					0.27–0.98					2.1–4.2
Black (Turkish coast)	2011	[84]	7–579	0.41–4.45	0.17–1.81	2.0–33.9	38–289	0.59–1.91	1.7–17.0	8.2–80.1	16–163	5.3–18.3			BDL–0.248	0.23–1.93	BDL–1.06				BDL–2.71
S Black (Sinop, Turkey)	2013	[72]	<0.5–6.9				29–37			0.7–1.0	35–47	2.3–4.1				0.4–0.77				<0.05	0.15–0.70
SW Black (Igneada, Turkey)	2012–2013	[85]														0.07–0.11				0.03–0.06	0.14–0.21
SW Black (Bulgarian coast) ^a^	2011	[86]				9.42–13.2						15.7–20.5				0.33–0.68				0.6–2.4	0.84–1.4
SW Black (Varna, Bulgaria) ^a^	2016	[49]														1.31–3.76				0.084–0.18	1.19–3.15
W Black (Romanian coast) ^a^	2011–2012	[87]			1.2–6.8				4.9–8.0	20.6–35.0						1.5–3.2					0.5–10.3
Marmara (Turkish coast)	2011	[84]	54–850	0.37–13.07	0.2–3.47	6.6–14.8	89–403	0.54–1.46	1.3–4.2	3.9–89.4	27–243	5.6–14.9			BDL–0.14	0.09–1.05	BDL–1.96				BDL–22.9
Marmara (Tekirdag, Turkey)	2012	[88]			BDL–2.46				<0.33–2.46		77–89	<0.33–2.79				<0.33–0.75				<0.1–<0.33	2.7–9.2
Levantine (Turkish coast)	2011	[84]	55–480	0.54–1.22	5.88–6.88	14.8–34.9	322–1060	1.0–1.42	6.7–12.3	15.1–172.5	281–340	6.1–9.3			0.46–5.1	1.43–2.36	0.225–0.423				8.36–20.04
Aegean (Turkish coast)	2011	[84]	206–730	0.65–2.32	2.17–5.18	9.1–16.5	192–440	0.69–1.76	2.6–9.2	6.4–11.0	183–441	7.2–14.0			0.11–0.36	0.44–3.32	0.123–0.163				0.06–12.58
Aegean (Saronic gulf, Greece)	?	[89]			1.7–8.1	7.2–60.7	82–1686		4.0–5.5	5.1–53.1	215–323					0.14–5.36					

^a^ Transformed from wet to dry weight basis.

**Table 6:** Element contents (min–max) in soft tissues of the sea snail *Rapana venosa* collected in different areas of the Marmara and Black Seas. Notations, emphases and shadings mean the same as in Table 5.

Sea (Area)	Year	Reference	Li	Al	Cr	Mn	Fe	Co	Ni	Cu	Zn	As	Cd	Hg	Pb
NW Black (Crimea, Russia)	2017	This work	BDL–61	440–38,200	**0.66–3.53**	2.8–13.4	**273–1820**	*0.05–0.12*	0.2–3.2	19.4–39.3	**124–254**	**28.7–51.1**	**5.8–14.8**	**0.65–2.91**	**BDL–10.7**
SE Black (Perşembe and Rize, Turkey)	1997–1998	[90]			<0.06–1.45	4.45–6.80	87–170	<0.05–0.7	1.17–3.74	47.44–72.20	73.3–200.9		2.01–30.69		<0.05
SE Black (Turkish coast)	?	[48]			1.0–1.6	5.63–15.8			0.79–1.1	56.7–92.8	51.5–106.4		3.9–7.7		0.88–4.3
S Black (Sinop, Turkey) ^a^	1997	[91]				1.5–4.7	75–262		1.5–25.0	5.3–35.6	50–338		0.1–0.97		0.36–2.0
S Black (Sinop, Turkey) ^a^	2013	[72]		<0.5			38–40			7.3–12.3	22–27	3.8–6.7	2.88–4.38	<0.05	<0.05
SW Black (R Feneri and Amasra, Turkey)	1997–1998	[90]			<0.06–0.73	3.90–10.01	134–550	<0.06–0.5	<0.01–3.74	36.19–41.02	230.4–255.9		15.59–41.13		<0.05
SW Black (Thrace coast, Turkey) ^a^	2012	[92]			0.1–0.2								0.1–1.6	0.4–0.7	0.1–0.7
SW Black (Igneada, Turkey)	2012–2013	[85]											0.08–0.14	0.18–0.44	0.09–0.18
SW Black (Varna Bay, Bulgaria)		[93]	BDL–60		BDL–19.0	231–283	BDL–1.2	0.2–5.0	1.8–18.0	16–44	6.4–95.0	BDL–6.8	0.5–1.0	BDL–0.3	15.3–33.0
SW Black (Bulgarian coast) ^a^		[86]				1.1–2.1						9.68–18.3	0.02–0.04	0.35–0.48	0.53–1.4
SW Black (Varna Bay, Bulgaria) ^a^		[49]											0.3–13.2	0.13–0.17	0.084–0.39
N Marmara (Turkish coast)		[94]								21.6–49.3	18–52		BDL–0.08		0.52–1.25

^a^ Transformed from wet to dry weight basis.

**Table 7:** Element contents (min–max) in soft tissues of the oyster *Crassostrea gigas* collected in different areas of the Atlantic basin. Notations, emphases and shadings mean the same as in Table 5.

Sea/Ocean (Area)	Year	Reference	Al	Cr	Mn	Fe	Ni	Cu	Zn	As	Se	Ag	Cd	Sn	Hg	Tl	Pb
N Black (Crimea, Russia)	2017	This work	**269–2287**	*0.16–0.20*	*5.2–5.6*	*200–220*	*0.52–0.53*	870–1455	**4070–5910**	*13.7–14.1*	6.2–6.8	8.7–112	**2.7–3.3**	BDL–0.025	**0.23–0.31**	0.003–0.005	0.3–4.1
W Adriatic (Italy)^a^	2014	[95]	138–1055	0.49–3.3	75–775	287–1355	1.2–2.2	301–11,550	1355–7265	21–83	5.3–12.0		1.6–10.4	<0.09–0.84	<0.09	<0.09	0.71–4.2
N, NW Adriatic (Italy)^a^	2014	[95]	76–1016	0.68–10.4	0.9–152	389–5756	1.1–7.3	42–4652	140–18660	13–147	0.09–13		0.62–7.2	<0.09–1.4	<0.09–3.2	<0.09	0.81–7.2
E Tyrrhenian (Orbello, Italy) ^a^	2014	[95]	177–623	0.02–1.6	75–149	348–748	1.2–2.6	676–1221	1800–4600	58–76	5.7–9.0		0.80–1.3	<0.09	2.1–2.7	<0.09	1.5–2.1
W Mediterranean (Sardinia, Italy) ^a^	2008–2012	[96]											0.66–1.60		0.08–0.16		1.24–4.52
W Mediterranean (Ebro Delta, Spain)	2008–2009	[97]						38.83–98.73	562–1125	0.001–0.0021			0.50–1.32		0.12–0.27		0.26–0.78
E Atlantic (Brittany, France) ^a^	2010	[98]						2.5–137	43.7–795				0.1–1.0				0.2–3.9
E Atlantic (Normandy, France)	2012–2013	[99]						30–1025	560–1800	19–42			0.94–2.76				0.83–2.37
English Channel, Helford estuary (Cornwall, England)	1972	[100]			12–36	120–800	2.0–7.3	100–790	1000–5400				1.3–4.7				4.1–14.0
SW North Sea (England)	2012	[101]						286–516	1663.7–2600				2.1–2.4				0.7–1.4
W Atlantic (Georgia, USA) ^b^	?	[79]			24–51	233–738		48–261	1500–5700			28–82					

^a^ Transformed from wet to dry weight basis. ^b^ Measured in soft tissues of *C. virginica*.

One can also observe the specific character of the regional spatial variability of the element contents. Increased trace element levels are observed in the mollusks from the western (Bulgaria and Romania), northwestern (Crimea) and southeastern (Giresun) coasts of the Black Sea and from the Marmara, Levantine, and Aegean seas. The increased levels in the Black Sea are likely related to the riverine discharge transporting sediments that can contain large amounts of trace elements. Rivers are believed to be the main source of the heavy metal pollution in the Black Sea [102,103,104]. The waters of the western and northwestern Black Sea are freshened by two major rivers in the region, the Danube and the Dnieper, and increased levels of heavy metals were indeed observed near the Danube mouth [105]. The southeastern peak of trace elements in the Black Sea mollusks is likely due to the inflow of wastewater effluents from mines in the northeast of Turkey [102,106,107], and both the seawater and sediments in this area are tangibly polluted with heavy metals [102]. The increased levels of trace elements in the mollusks from the Marmara, Levantine, and Aegean seas may be attributed to the heavy ship traffic and intense activity of major regional seaports (Istanbul, Beirut, and Athens).

## 5. Conclusions

To assess the inorganic micronutrient capacity and the trace element poisoning hazard to humans, we performed a comprehensive analysis of trace elements in soft tissues of three commercially important edible mollusks (the mussel *Mytilus galloprovincialis*, the sea snail *Rapana venosa*, and the oyster *Crassostrea gigas*) from the Crimean coastal zone in the Black Sea. For the first time, an unprecedented number of elements (72) in the mollusks were quantified. The contents of these elements used in the hierarchical and centroid-based cluster analysis has allowed separating the elements in several non-overlapping optimal clusters that are made up of 1 group of macrominerals, 1–2 groups of trace elements and 1–2 groups of ultratrace elements. The numbers of elements in the groups fitted by a quadratic equation have enabled introducing the element accumulation universality index as the reciprocal quadratic term, which indicates how many elements can be most efficiently accumulated by the mollusk. The element accumulation universality index has been found to increase in the following order: oyster–sea snail–mussel. For the three species under study, the numbers of the most efficiently accumulated elements in each species are linearly correlated with the accumulation universality indices with a high degree of linearity. It is important to study in the future whether this linearity will be observed also for other mollusk species. This research offers a methodology to estimating the universality of accumulating other materials, e.g., pesticides, by the mollusks. In perspective, this approach can be extended to other phyla of marine biota.

The similarities of the essential element accumulation in the two bivalve species (the mussel and the oyster) were demonstrated in the ternary diagram and by comparison with the element contents in the mollusks from other areas.

From the comparison with the contamination-indicative concentrations and element contents from other reports, it was concluded that the sea area used for the mollusk farming is likely contaminated with such heavy metals as Zn, Cd, and Hg. The origin of this contamination is not clear and requires further research. A possible major source of the elements in the sea area near the western coast of Crimea can be the Dnieper River flowing into the northwestern part of the Black Sea. In support of this statement, it was observed that the locations of the Danube discharge and the metal-polluted rivers in the northeast of Turkey correlate with the increased levels of trace elements in the mollusks and environment.

The contents of some elements in the mollusks under study were shown to exceed the maximum permissible levels from the EU and/or Russian regulations (e.g., cadmium in the sea snail), and it was interesting to assess whether or not the daily element intakes due to the mollusk consumption by the Crimean population in 2017 exceeded the tolerable values according to the USEPA recommendations. For this assessment, we presented a novel original approach to estimating the mollusk consumption rate based on the obsolete data from the FAO database plotted against the product of the average salary (or, equivalently, gross domestic product at purchasing power parity), coastline length, and population density; this approach can be extended to any particular territory with access to the sea. From this analysis, at least two groups of countries with different consumption rate patterns were identified, and accordingly, two different consumption rate scenarios have emerged. From the expected rates in both scenarios, the mollusk consumption did not pose any health risks to the population of Crimea. However, using the maximum consumption in the pessimistic scenario, the long-term toxic action of the trace elements from the mussel and sea snail could not be ruled out within the 95% probability. In view of the permanent changes in the aquatic environment, physiological state of mollusks, and socioeconomic and demographic situation in the society, this result underscores the urgency of regular spatiotemporal monitoring of toxic trace element levels in the seafood.

## Figures and Tables

**Table 1:** Trace element contents in European Reference Material ERM^®^-CE278k: Mean value ± uncertainty at the 95% confidence level.

	Certified Value (μg·g^−1^ d.w.)	Observed Value (μg·g^−1^ d.w.), *n* = 5
As	6.7 ± 0.4	5.7 ± 1.6
Cd	0.336 ± 0.025	0.37 ± 0.18
Co	0.21	0.21 ± 0.07
Cr	0.73 ± 0.22	0.85 ± 0.16
Cu	5.98 ± 0.27	5.60 ± 0.35
Fe	161 ± 8	156 ± 3
Hg	0.071 ± 0.007	0.067 ± 0.004
Mn	4.88 ± 0.24	4.97 ± 0.61
Ni	0.69 ± 0.15	0.60 ± 0.23
Pb	2.18 ± 0.18	2.28 ± 0.55
Rb	2.46 ± 0.16	2.23 ± 0.14
Se	1.62 ± 0.12	1.65 ± 0.34
Sr	19.0 ± 1.2	20.5 ± 4.8
Zn	71 ± 4	65 ± 6

**Table 2:** Mean characteristics of seawater at the sampling site in April–May 2017: temperature T (°C), salinity S (psu or ‰), dissolved oxygen O_2_ (mL·L^−1^), pH value, five-day biochemical oxygen demand BOD_5_ (mg O_2_·L^−1^), alkaline permanganate oxidizability Ox (mg O_2_·L^−1^), nitrite NO_2_, nitrate NO_3_, ammonium NH_4_, phosphate PO_4_, silicate Si, organic phosphorus P_org_, and organic nitrogen N_org_ (all in μM).

T	S	O_2_	pH	BOD_5_	Ox	NO_2_	NO_3_	NH_4_	PO_4_	Si	P_org_	N_org_
11.4	17.99	7.09	8.28	1.49	2.49	0.08	1.1	0.59	0.09	1.59	0.18	32

**Table 3:** Trace element contents in soft tissues of three mollusk species harvested in the mussel-and-oyster farm area near Sevastopol (northwestern Black Sea). Dark shading: in g·kg^−1^, light shading: in in mg·kg^−1^, no shading: in μg·kg^−1^ d.w. In parentheses are the maximum permissible levels [56,57] and/or contamination-indicative concentrations (CIC) [58] for each species. Ref: reference contents (CIC and action levels [59], AL) for mussels and oysters (CIC) or all types of edible mollusks (AL). MPI: metal pollution index [63]. AUI: accumulation universality index.

	*M. galloprovincialis*	*R. venosa*	*C. gigas*	Ref
Li	100 ± 20	28 ± 18	227 ± 74	
Be	7.5 ± 1.4	6.8 ± 5.9	26.1 ± 14.5	
B	16.6 ± 1.6	30 ± 17	17 ± 1	
F	1506 ± 180	236 ± 4	198 ± 10	
Na	11.5 ± 1.6	7.6 ± 2.0	27 ± 3	
Mg	2.2 ± 0.3	10.1 ± 1.2	3.5 ± 0.2	
Al	3.0 ± 2.2	13.1 ± 12.6	1.28 ± 1.01	
Si	0.68 ± 0.02	1.52 ± 0.68	0.71 ± 0.02	
P	4.4 ± 0.6	6.0 ± 0.1	7.6 ± 0.7	
K	7.1 ± 0.7	13.6 ± 3.6	9.23 ± 0.04	
Ca	0.90 ± 0.12	2.86 ± 0.60	5.8 ± 2.8	
Sc	12.0 ± 1.6	0.21 ± 0.02	0.36 ± 0.12	
Ti	96 ± 13	3.1 ± 2.7	0.60 ± 0.06	
V	1.14 ± 0.18	0.55 ± 0.21	0.32 ± 0.07	
Cr	2.61 ± 0.34	2.06 ± 0.83	0.18 ± 0.02	2.5 ^c^, 13 ^d^
Mn	6.3 ± 1.4	8.3 ± 3.1	5.4 ± 0.2	
Fe	197 ± 26	821 ± 500	210 ± 10	
Co	644 ± 112	91 ± 21	52 ± 6	
Ni	5.2 ± 0.8	1.47 ± 0.89	0.52 ± 0.00	3.4 ^c^, 80 ^d^
Cu	7.2 ± 1.1 (189 ^a^, 10 ^c^)	28 ± 6 (125 ^a^)	1161 ± 293 (284 ^a^, 300 ^c^)	
Zn	308 ± 59 (1260 ^a^, 200 ^c^)	181 ± 38 (836 ^a^)	4990 ± 921 (1894 ^a^, 4000 ^c^)	
Ga	0.55 ± 0.21	0.51 ± 0.46	0.06 ± 0.06	
Ge	1.12 ± 0.13	0.12 ± 0.05	0.06 ± 0.01	
As	15.1 ± 2.6	37 ± 7	13.9 ± 0.2	16 ^c^, 86 ^d^
Se	8.8 ± 1.1	5.1 ± 0.5	6.5 ± 0.3	
Br	61 ± 6	423 ± 88	122 ± 6	
Rb	3.2 ± 0.3	3.5 ± 0.5	2.9 ± 0.2	
Sr	35 ± 11	129 ± 90	51 ± 13	
Y	72 ± 18	9.0 ± 3.8	35 ± 22	
Zr	150 ± 26	284 ± 231	12 ± 12	
Nb	19 ± 4	17 ± 7	5.5 ± 0.1	
Mo	858 ± 136	310 ± 115	192 ± 71	
Ru	5.8 ± 1.5	0.95 ± 0.95	0.54 ± 0.54	
Rh	37 ± 14	6.3 ± 5.6	42 ± 13	
Pd	533 ± 157	65 ± 65	107 ± 38	
Ag	0.48 ± 0.09 (0.75 ^c^)	7.8 ± 2.3	60 ± 52 (5 ^c^)	
Cd	3.88 ± 0.82 (12.6 ^a^, 6.3 ^b^)	10.0 ± 2.6 (8.4 ^a^, 4.2 ^b^)	3.0 ± 0.3 (18.94 ^a^, 9.47 ^b^)	3.7 ^c^, 4 ^d^
In	17 ± 3	1.71 ± 0.88	25 ± 18	
Sn	249 ± 67	96 ± 70	12 ± 12	
Sb	86 ± 18	52 ± 13	4.3 ± 4.3	
Te	19 ± 3	6.9 ± 2.6	7.2 ± 1.9	
I	13.9 ± 0.9	181 ± 62	24 ± 2	
Cs	36 ± 9	9.4 ± 5.1	18 ± 13	
Ba	3.8 ± 1.4	4.7 ± 3.5	228 ± 222	
La	2.54 ± 0.52	0.49 ± 0.24	0.13 ± 0.13	
Ce	137 ± 31	65 ± 38	54 ± 20	
Pr	13 ± 3	4.3 ± 3.3	12 ± 11	
Nd	70 ± 16	20 ± 15	17 ± 11	
Sm	14 ± 3	5.1 ± 3.4	6.8 ± 0.1	
Eu	53 ± 19	3.5 ± 2.6	7.7 ± 5.8	
Gd	13 ± 3	7.0 ± 4.8	8.9 ± 1.6	
Tb	22 ± 4	0.91 ± 0.52	13 ± 6	
Dy	11 ± 3	6.2 ± 4.9	12 ± 3	
Ho	2.24 ± 0.59	1.73 ± 1.35	10 ± 1	
Er	18 ± 5	4.6 ± 2.3	12 ± 5	
Tm	2.97 ± 2.09	0.87 ± 0.68	8.4 ± 2.0	
Yb	7.4 ± 1.5	6.0 ± 3.2	9.8 ± 7.6	
Lu	6.2 ± 1.1	0.93 ± 0.84	18 ± 12	
Hf	159 ± 40	66 ± 40	23 ± 4	
Ta	12 ± 2	10 ± 4	0.13 ± 0.13	
W	277 ± 73	100 ± 26	38 ± 38	
Re	1.14 ± 0.35	5.7 ± 3.1	0.08 ± 0.08	
Os	94 ± 20	28 ± 12	4.8 ± 4.4	
Ir	72 ± 19	18 ± 5	6.0 ± 4.3	
Pt	50 ± 7	38 ± 16	8.7 ± 1.7	
Au	278 ± 45	115 ± 32	182 ± 49	
Hg	0.96 ± 0.31 (1.3 ^a^, 3.1 ^b^)	1.67 ± 0.66 (0.84 ^a^, 2.1 ^b^)	0.27 ± 0.04 (1.89 ^a^, 4.74 ^b^)	0.23 ^c^
Tl	16 ± 2	5.9 ± 4.3	3.9 ± 0.9	
Pb	1.65 ± 0.49 (63 ^a^, 9.4 ^b^)	3.5 ± 1.5 (42 ^a^, 6.3 ^b^)	2.18 ± 1.90 (94.7 ^a^, 14.2 ^b^)	3.2 ^c^, 1.7 ^d^
Bi	186 ± 34	3.6 ± 3.6	33 ± 20	
Th	132 ± 15	23 ± 12	258 ± 216	
U	67 ± 19	138 ± 94	54 ± 4	
MPI ^e^	1.51	1.54	1.19	
AUI	2.63	1.18	0.34	

^a^ Maximum permissible level from [56]. ^b^ Maximum permissible level from [57]. ^c^ Contamination-indicative content, from [58]. ^d^ USFDA action levels, from [59]. ^e^ Calculated for metals with oral reference doses listed in [54].

**Table 4:** Target hazard quotients (THQ) calculated for the pessimistic (THQ_1_) and optimistic (THQ_2_) scenarios using the oral reference doses [54]. HI: hazard index. THQ are presented as expected and (in parentheses) maximum values. The minimum values in all the cases are 0. Bold emphasis: THQ > 10^−1^ and HI > 1.

	THQ_1_ (Mussel)	THQ_2_ (Mussel)	THQ_1_ (Sea Snail)	THQ_2_ (Sea Snail)	THQ_1_ (Oyster)	THQ_2_ (Oyster)
Li	3.1∙10^−4^ (1.4∙10^−3^)	1.7∙10^−4^ (4.7∙10^−4^)	8.8∙10^−5^ (3.6∙10^−4^)	4.2∙10^−5^ (1.2∙10^−4^)	2.9∙10^−4^ (1.2∙10^−3^)	1.4∙10^−4^ (3.9∙10^−4^)
Be	2.4∙10^−5^ (1.1∙10^−4^)	1.2∙10^−5^ (3.6∙10^−5^)	2.1∙10^−5^ (8.7∙10^−5^)	1.0∙10^−5^ (2.9∙10^−5^)	3.3∙10^−5^ (1.3∙10^−4^)	1.6∙10^−5^ (4.5∙10^−5^)
B	5.2∙10^−4^ (2.3∙10^−3^)	2.8∙10^−4^ (7.9∙10^−4^)	9.4∙10^−4^ (3.8∙10^−3^)	4.5∙10^−4^ (1.3∙10^−3^)	2.2∙10^−4^ (8.8∙10^−4^)	1.0∙10^−4^ (2.9∙10^−4^)
F	**1.6∙10^−1^ (7.1∙10^−1^)**	8.3∙10^−2^ (**2.4∙10^−1^**)	2.5∙10^−2^ (**1.0∙10^−1^**)	1.2∙10^−2^ (3.4∙10^−2^)	8.4∙10^−3^ (3.4∙10^−2^)	4.0∙10^−3^ (1.1∙10^−2^)
Al	1.9∙10^−2^ (8.5∙10^−2^)	9.9∙10^−3^ (2.8∙10^−2^)	8.2∙10^−2^ (**3.3∙10^−1^**)	3.9∙10^−2^ (**1.1∙10^−1^**)	3.2∙10^−3^ (1.3∙10^−2^)	1.5∙10^−3^ (4.4∙10^−3^)
V	1.4∙10^−3^ (6.5∙10^−3^)	7.6∙10^−4^ (2.2∙10^−3^)	6.9∙10^−4^ (2.8∙10^−3^)	3.3∙10^−4^ (9.4∙10^−4^)	1.6∙10^−4^ (6.6∙10^−4^)	7.7∙10^−5^ (2.2∙10^−4^)
Mn	2.8∙10^−4^ (1.3∙10^−3^)	1.5∙10^−4^ (4.3∙10^−4^)	3.7∙10^−4^ (1.5∙10^−3^)	1.8∙10^−4^ (5.1∙10^−4^)	9.8∙10^−5^ (4.0∙10^−4^)	4.7∙10^−5^ (1.3∙10^−4^)
Fe	1.8∙10^−3^ (8.0∙10^−3^)	9.3∙10^−4^ (2.7∙10^−3^)	7.4∙10^−3^ (3.0∙10^−2^)	3.5∙10^−3^ (1.0∙10^−2^)	7.6∙10^−4^ (3.1∙10^−3^)	3.6∙10^−4^ (1.0∙10^−3^)
Co	1.4∙10^−2^ (6.1∙10^−2^)	7.1∙10^−3^ (2.0∙10^−2^)	1.9∙10^−3^ (7.8∙10^−3^)	9.1∙10^−4^ (2.6∙10^−3^)	4.4∙10^−4^ (1.8∙10^−3^)	2.1∙10^−4^ (6.0∙10^−4^)
Ni	1.6∙10^−3^ (7.4∙10^−3^)	8.6∙10^−4^ (2.5∙10^−3^)	4.6∙10^−4^ (1.9∙10^−3^)	2.2∙10^−4^ (6.3∙10^−4^)	6.6∙10^−5^ (2.7∙10^−4^)	3.1∙10^−5^ (9.0∙10^−5^)
Cu	1.1∙10^−3^ (5.1∙10^−3^)	6.0∙10^−4^ (1.7∙10^−3^)	4.4∙10^−3^ (1.8∙10^−2^)	2.1∙10^−3^ (6.0∙10^−3^)	7.4∙10^−2^ (**3.0∙10^−1^**)	3.5∙10^−2^ (**1.0∙10^−1^**)
Zn	6.5∙10^−3^ (2.9∙10^−2^)	3.4∙10^−3^ (9.7∙10^−3^)	3.8∙10^−3^ (1.5∙10^−2^)	1.8∙10^−3^ (5.2∙10^−3^)	4.2∙10^−2^ (**1.7∙10^−1^**)	2.0∙10^−2^ (5.7∙10^−2^)
As	2.8∙10^−2^ (**1.3∙10^−1^**)	1.5∙10^−2^ (4.3∙10^−2^)	5.4∙10^−2^ (**2.2∙10^−1^**)	2.6∙10^−2^ (7.4∙10^−2^)	1.1∙10^−2^ (4.3∙10^−2^)	5.0∙10^−3^ (1.4∙10^−2^)
Se	1.1∙10^−2^ (5.0∙10^−2^)	5.8∙10^−3^ (1.7∙10^−2^)	6.4∙10^−3^ (2.6∙10^−2^)	3.1∙10^−3^ (8.7∙10^−3^)	3.3∙10^−3^ (1.3∙10^−2^)	1.6∙10^−3^ (4.5∙10^−3^)
Sr	3.7∙10^−4^ (1.7∙10^−3^)	1.9∙10^−4^ (5.5∙10^−4^)	1.4∙10^−3^ (5.5∙10^−3^)	6.4∙10^−4^ (1.8∙10^−3^)	2.2∙10^−4^ (8.8∙10^−4^)	1.0∙10^−4^ (2.9∙10^−4^)
Zr	1.2∙10^−2^ (5.3∙10^−2^)	6.2∙10^−3^ (1.8∙10^−2^)	2.2∙10^−2^ (9.1∙10^−2^)	1.1∙10^−2^ (3.0∙10^−2^)	3.8∙10^−4^ (1.5∙10^−3^)	1.8∙10^−4^ (5.2∙10^−4^)
Mo	1.1∙10^−3^ (4.9∙10^−3^)	5.7∙10^−4^ (1.6∙10^−3^)	3.9∙10^−4^ (1.6∙10^−3^)	1.9∙10^−4^ (5.3∙10^−4^)	9.7∙10^−5^ (4.0∙10^−4^)	4.6∙10^−5^ (1.3∙10^−4^)
Ag	6.0∙10^−4^ (2.7∙10^−3^)	3.2∙10^−4^ (9.1∙10^−4^)	9.8∙10^−3^ (4.0∙10^−2^)	4.7∙10^−3^ (1.3∙10^−2^)	3.0∙10^−2^ (**1.2∙10^−1^**)	1.4∙10^−2^ (4.1∙10^−2^)
Cd	2.4∙10^−2^ (**1.1∙10^−1^**)	1.3∙10^−2^ (3.7∙10^−2^)	6.3∙10^−2^ (**2.6∙10^−1^**)	3.0∙10^−2^ (8.6∙10^−2^)	7.6∙10^−3^ (3.1∙10^−2^)	3.6∙10^−3^ (1.0∙10^−2^)
Sn	2.6∙10^−6^ (1.2∙10^−5^)	1.4∙10^−6^ (3.9∙10^−6^)	1.0∙10^−6^ (4.1∙10^−6^)	4.8∙10^−7^ (1.4∙10^−6^)	5.1∙10^−8^ (2.1∙10^−7^)	2.4∙10^−8^ (6.9∙10^−8^)
Sb	1.4∙10^−3^ (6.1∙10^−3^)	7.1∙10^−4^ (2.0∙10^−3^)	8.2∙10^−4^ (3.3∙10^−3^)	3.9∙10^−4^ (1.1∙10^−3^)	2.7∙10^−5^ (1.1∙10^−4^)	1.3∙10^−5^ (3.7∙10^−5^)
I	8.7∙10^−3^ (3.9∙10^−2^)	4.6∙10^−3^ (1.3∙10^−2^)	**1.1∙10^−1^ (4.6∙10^−1^)**	5.4∙10^−2^ (**1.5∙10^−1^**)	6.1∙10^−3^ (2.5∙10^−2^)	2.9∙10^−3^ (8.3∙10^−3^)
Ba	1.2∙10^−4^ (5.4∙10^−4^)	6.3∙10^−5^ (1.8∙10^−4^)	1.5∙10^−4^ (6.0∙10^−4^)	7.0∙10^−5^ (2.0∙10^−4^)	2.9∙10^−3^ (1.2∙10^−2^)	1.4∙10^−3^ (3.9∙10^−3^)
La	**3.2∙10^−1^ (1.4∙10^0^)**	**1.7∙10^−1^ (4.8∙10^−1^)**	6.2∙10^−2^ (**2.5∙10^−1^**)	2.9∙10^−2^ (8.4∙10^−2^)	6.6∙10^−3^ (2.7∙10^−2^)	3.1∙10^−3^ (9.0∙10^−3^)
W	2.2∙10^−3^ (9.8∙10^−3^)	1.1∙10^−3^ (3.3∙10^−3^)	7.9∙10^−4^ (3.2∙10^−3^)	3.7∙10^−4^ (1.1∙10^−3^)	1.2∙10^−4^ (4.9∙10^−4^)	5.7∙10^−5^ (1.6∙10^−4^)
Hg	2.0∙10^−2^ (9.1∙10^−2^)	1.1∙10^−2^ (3.0∙10^−2^)	3.5∙10^−2^ (**1.4∙10^−1^**)	1.7∙10^−2^ (4.8∙10^−2^)	2.3∙10^−3^ (9.3∙10^−3^)	1.1∙10^−3^ (3.1∙10^−3^)
Tl	1.0∙10^−2^ (4.5∙10^−2^)	5.3∙10^−3^ (1.5∙10^−2^)	3.7∙10^−3^ (1.5∙10^−2^)	1.8∙10^−3^ (5.1∙10^−3^)	9.9∙10^−4^ (4.0∙10^−3^)	4.7∙10^−4^ (1.3∙10^−3^)
Pb	5.2∙10^−3^ (2.3∙10^−2^)	2.7∙10^−3^ (7.8∙10^−3^)	1.1∙10^−2^ (4.5∙10^−2^)	5.2∙10^−3^ (1.5∙10^−2^)	2.8∙10^−3^ (1.1∙10^−2^)	1.3∙10^−3^ (3.8∙10^−3^)
U	2.1∙10^−3^ (9.5∙10^−3^)	1.1∙10^−3^ (3.2∙10^−3^)	4.3∙10^−3^ (1.8∙10^−2^)	2.1∙10^−3^ (5.9∙10^−3^)	6.9∙10^−4^ (2.8∙10^−3^)	3.3∙10^−4^ (9.3∙10^−4^)
HI	0.65 (**2.93**)	0.34 (0.98)	0.52 (**2.10**)	0.25 (0.70)	0.20 (0.83)	0.10 (0.28)

## Data Availability

The data used in this study are available from the corresponding author on reasonable request.

## Supplementary Materials

The following are available online at https://www.mdpi.com/article/10.3390/foods10102313/s1, Figure S1: Finding the optimum number of non-overlapping clusters from the logarithm of summed distances to centroids (Υ(*k*), dots) using two piecewise linear fits and minimum in the mean summed residuals (crosses) for (a) *M. galloprovincialis*, (b) *R. venosa*, and (c) *C. gigas*; Table S1: Parameters used for finding correlations between the mollusk consumption rate (CR) and GDP·PD·L where GDP is the gross domestic product per capita at purchasing power parity, PD is population density, and L is the coastline length (see Section 3.3); Table S2: Statistical comparison of the linear fits for all selected countries and those with the lower consumption rates correlated with GDP·PD·L; Table S3: Tolerable daily intakes (in mg·kg^−1^_body weight_·day^−1^) of the elements set by the US Environmental Protection Agency (RfD_o_), World Health Organization (PTDI), and European Food Safety Authority (UDI) and their estimated daily intakes (EDI, in mg·kg^−1^_body weight_·day^−1^) with the three edible mollusk species according to the pessimistic (EDI_1_) and optimistic (EDI_2_) scenarios. EDI are presented as expected and (in parentheses) maximum values. The minimum values in all the cases are 0. The estimated daily intakes exceeding any of the tolerable levels are emphasized in bold.
