# Supplementary material for: Element Contents in Three Commercially Important Edible Mollusks Harvested off the Southwestern Coast of Crimea (Black Sea) and Assessment of Human Health Risks from Their Consumption
Source: Foods. 2021 Sep 29;10(10):2313. doi: 10.3390/foods10102313 (PMC8534638; doi:10.3390/foods10102313)
Supplement: Supplementary file 1 [file foods-10-02313-s001.zip › foods-1325939-supplementary.pdf]

Supplementary Material

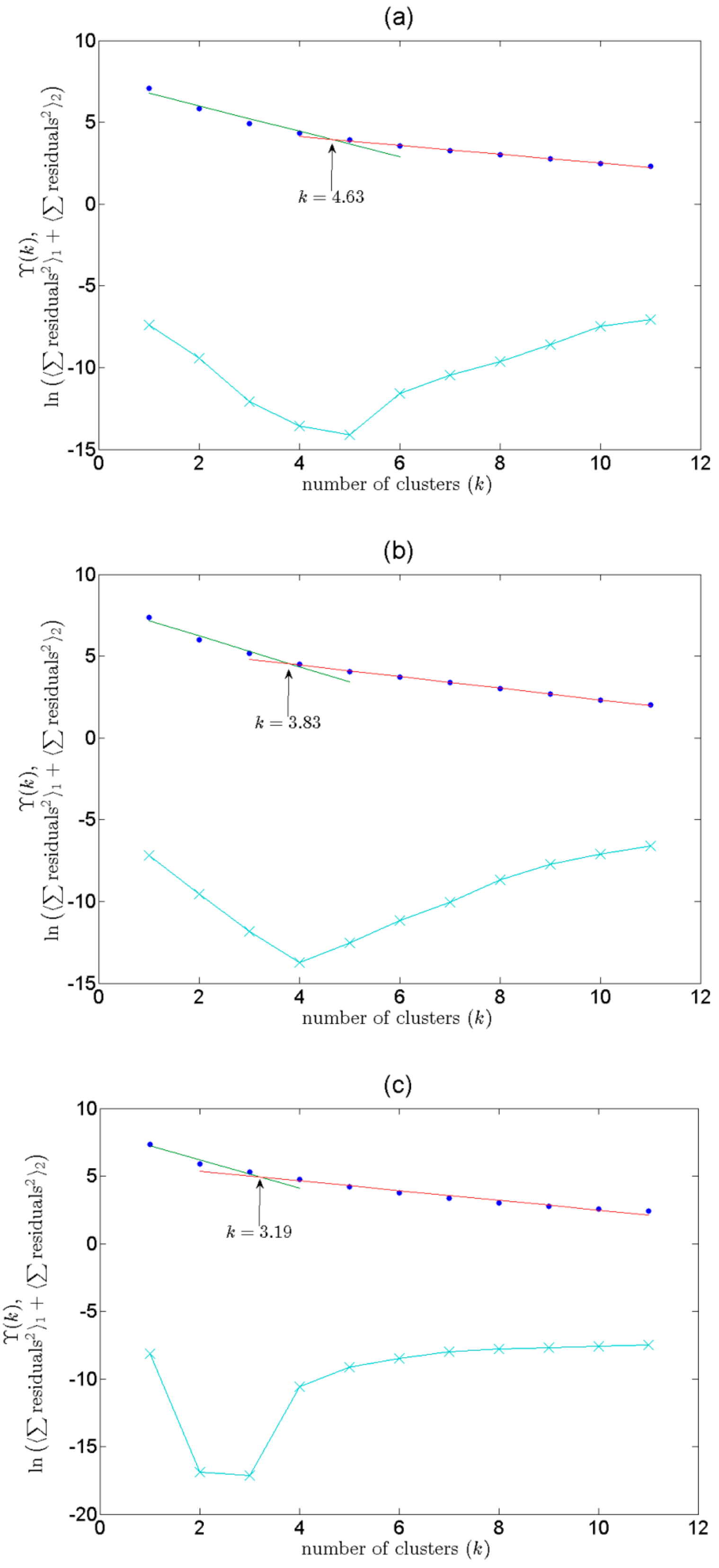

Figure S1. Finding the optimum number of non-overlapping clusters from the logarithm of summed distances to centroids ($\Upsilon(k)$, dots) using two piecewise linear fits and minimum in the mean summed residuals (crosses) for **(a)** *M. galloprovincialis*, **(b)** .*R. venosa*, and **(c)** *C. gigas*.

Table S1. Parameters used for finding correlations between the mollusk consumption rate (CR) and GDP·PD·L where GDP is the gross domestic product per capita at purchasing power parity, PD is population density, and L is the coastline length (see Section 3.3).

	**L, km**	**GDP, $**	**PD, km^{-2}**	**GDP·PD·L, ×10^{9} $·year^{-1}·km^{-1}**	**CR, g·capita^{-1}·day^{-1}**
Belgium	66.5	45498.35	363.25	1.10	11.254
Bulgaria	354	7332.21	67.72	0.18	0.474
China	14500	5473.68	142.63	11.32	22.666
Croatia	1777.3	14006.45	76.50	1.90	3.314
Cyprus	648	30434.12	121.52	2.40	2.314
Denmark	7314	59531.40	134.47	58.55	4.574
Egypt	2450	2852.28	85.04	0.59	0.202
France	4853	41894.42	119.34	24.26	19.924
Georgia	310	3824.65	65.80	0.08	0.41
Greece	13676	25332.50	85.87	29.75	2.468
Israel	273	32179.78	359.01	3.15	0.834
Italy	7600	36456.53	202.30	56.05	11.806
Lebanon	245	7734.64	516.55	0.98	0.974
Malaysia	4675	9704.00	87.13	3.95	6.786
Malta	252.8	22667.34	1305.78	7.48	11.684
Montenegro	293.5	6903.55	46.09	0.09	2.972
Portugal	1793	22191.47	115.01	4.58	11.678
Romania	256	8783.27	87.63	0.20	0.168
Russia	37653	12988.88	8.74	4.27	1.2
Slovenia	46.6	23887.72	101.85	0.11	2.166
South Korea	2413	23995.37	513.02	29.70	28.56
Spain	4964	30313.12	93.27	14.03	17.924
Thailand	3219	5362.06	132.15	2.28	6.118
Turkey	7200	11135.34	95.56	7.66	1.372
Ukraine	2782	3392.60	78.96	0.75	2.062
Vietnam	3444	1536.42	286.70	1.52	4.852
Crimea, 2017	945	5385.76	87.15	0.44	-

Table S2. Statistical comparison of the linear fits for all selected countries and those with the lower consumption rates correlated with GDP·PD·L.

Comparison of Fits	A (all CR)	B (lower CR)	Global (shared)
Null hypothesis			One curve for all data sets
Alternative hypothesis			Different curve for each data set
P value			<0.0001
Conclusion (alpha = 0.05)			Reject null hypothesis
Preferred model			Different curve for each data set
F (DFn, DFd)			45.07 (2, 28)
Different curve for each data set			
Best-fit values			
Slope	1.281	1.300	
Yintercept	0.9047	0.1679	
95% CI (profile likelihood)			
Slope	-0.06791 to 2.630	-0.8868 to 3.487	
Yintercept	0.7656 to 1.044	0.04595 to 0.2899	
Goodness of Fit			
Degrees of Freedom	17	11	
R squared	0.9172	0.4549	
Sum of Squares	94.71	107.7	
Sy.x	2.360	3.128	
One curve for all data sets			
Best-fit values			
Slope	0.3519	0.3519	0.3519
Yintercept	2.328	2.328	2.328
95% CI (profile likelihood)			
Slope	0.1907 to 0.5131	0.1907 to 0.5131	0.1907 to 0.5131
Yintercept	0.1268 to 4.529	0.1268 to 4.529	0.1268 to 4.529
Goodness of Fit			
Degrees of Freedom			30
R squared	0.5208	0.5476	0.3984
Sum of Squares	548.1	305.7	853.8
Sy.x			5.335
Constraints			
Slope	Slope is shared	Slope is shared	
Yintercept	Yintercept is shared	Yintercept is shared	
Number of points			
# of X values	28	28	
# Y values analyzed	19	13	

Table S3. Tolerable daily intakes (in mg·kg$^{-1}_{body\ weight}$·day^{-1}) of the elements set by the US Environmental Protection Agency (RfD$_o$), World Health Organization (PTDI), and European Food Safety Authority (UDI) and their estimated daily intakes (EDI, in mg·kg$^{-1}_{body\ weight}$·day^{-1}) with the three edible mollusk species according to the pessimistic (EDI$_1$) and optimistic (EDI$_2$) scenarios. EDI are presented as expected and (in parentheses) maximum values. The minimum values in all the cases are 0. The estimated daily intakes exceeding any of the tolerable levels are emphasized in bold.

	RfD$_o$	PTDI	UDI	EDI$_1$ (mussel)	EDI$_2$ (mussel)	EDI$_1$ (sea	EDI$_2$ (sea	EDI$_1$ (oyster)	EDI$_2$ (oyster)
Li	2E-3			6.3E-7 (2.8E-6)	3.3E-7 (9.5E-7)	1.8E-7 (7.2E-7)	8.4E-8 (2.4E-7)	5.8E-7 (2.3E-6)	2.7E-7 (7.8E-7)
Be	2E-3	2.9E-4		4.7E-8 (2.1E-7)	2.5E-8 (7.1E-8)	4.3E-8 (1.7E-7)	2.0E-8 (5.8E-8)	6.6E-8 (2.7E-7)	3.2E-8 (9.0E-8)
B	2E-1	2.7E-2	2.0E-2	1.0E-4 (4.7E-4)	5.5E-5 (1.6E-4)	1.9E-4 (7.7E-4)	9.0E-5 (2.6E-4)	4.3E-5 (1.8E-4)	2.1E-5 (5.9E-5)
F	6E-2		1.0E-1	9.5E-3 (4.3E-2)	5.0E-3 (1.4E-2)	1.5E-3 (6.0E-3)	7.1E-4 (2.0E-3)	5.0E-4 (2.0E-3)	2.4E-4 (6.8E-4)
Al	1E+0	2.9E-1	1.4E-1	1.9E-2 (8.5E-2)	9.9E-3 (2.8E-2)	8.2E-2 (3.3E-1)	3.9E-2 (1.1E-1)	3.2E-3 (1.3E-2)	1.5E-3 (4.4E-3)
V	5E-3			7.2E-6 (3.2E-5)	3.8E-6 (1.1E-5)	3.5E-6 (1.4E-5)	1.6E-6 (4.7E-6)	8.1E-7 (3.3E-6)	3.9E-7 (1.1E-6)
Mn	1.4E-1			4.0E-5 (1.8E-4)	2.1E-5 (6.0E-5)	5.2E-5 (2.1E-4)	2.5E-5 (7.1E-5)	1.4E-5 (5.6E-5)	6.5E-6 (1.9E-5)
Fe	7E-1	8.0E-1		1.2E-3 (5.6E-3)	6.5E-4 (1.9E-3)	5.2E-3 (2.1E-2)	2.5E-3 (7.0E-3)	5.3E-4 (2.2E-3)	2.5E-4 (7.3E-4)
Co	3E-4		2.3E-4	4.1E-6 (1.8E-5)	2.1E-6 (6.1E-6)	5.7E-7 (2.3E-6)	2.7E-7 (7.8E-7)	1.3E-7 (5.4E-7)	6.3E-8 (1.8E-7)
Ni	2E-2	3.1E-3	4.0E-4	3.3E-5 (1.5E-4)	1.7E-5 (4.9E-5)	9.2E-6 (3.8E-5)	4.4E-6 (1.3E-5)	1.3E-6 (5.4E-6)	6.3E-7 (1.8E-6)
Cu	4E-2	5.0E-1	7.1E-2	4.5E-5 (2.0E-4)	2.4E-5 (6.8E-5)	1.8E-4 (7.2E-4)	8.4E-5 (2.4E-4)	2.9E-3 (1.2E-2)	1.4E-3 (4.0E-3)
Zn	3E-1	1.0E+0	3.6E-1	1.9E-3 (8.7E-3)	1.0E-3 (2.9E-3)	1.1E-3 (4.6E-3)	5.4E-4 (1.5E-3)	1.3E-2 (5.1E-2)	6.0E-3 (1.7E-2)
As[a]	3E-4	2.1E-3[b]		8.5E-6 (3.8E-5)	4.5E-6 (1.3E-5)	1.6E-5 (6.6E-5)	7.8E-6 (2.2E-5)	3.2E-6 (1.3E-5)	1.5E-6 (4.3E-6)
Se	5E-3	5.7E-3	4.3E-3	5.5E-5 (2.5E-4)	2.9E-5 (8.3E-5)	3.2E-5 (1.3E-4)	1.5E-5 (4.4E-5)	1.6E-5 (6.7E-5)	7.8E-6 (2.2E-5)
Sr	6E-1	1.9E-2		2.2E-4 (9.9E-4)	1.2E-4 (3.3E-4)	8.1E-4 (3.3E-3)	3.9E-4 (1.1E-3)	1.3E-4 (5.3E-4)	6.2E-5 (1.8E-4)
Zr	8E-5			9.4E-7 (4.2E-6)	5.0E-7 (1.4E-6)	1.8E-6 (7.3E-6)	8.5E-7 (2.4E-6)	3.0E-8 (1.2E-7)	1.4E-8 (4.1E-8)
Mo	5E-3		1.0E-2	5.4E-6 (2.4E-5)	2.8E-6 (8.1E-6)	2.0E-6 (7.9E-6)	9.3E-7 (2.7E-6)	4.9E-7 (2.0E-6)	2.3E-7 (6.6E-7)
Ag	5E-3			3.0E-6 (1.4E-5)	1.6E-6 (4.6E-6)	4.9E-5 (2.0E-4)	2.3E-5 (6.7E-5)	1.5E-4 (6.2E-4)	7.2E-5 (2.1E-4)
Cd	1E-3	1.0E-3[b]	3.6E-4	2.4E-5 (1.1E-4)	1.3E-5 (3.7E-5)	6.3E-5 (2.6E-4)	3.0E-5 (8.6E-5)	7.6E-6 (3.1E-5)	3.6E-6 (1.0E-5)
Sn	6E-1	2.0E+0		1.6E-6 (7.0E-6)	8.3E-7 (2.4E-6)	6.0E-7 (2.5E-6)	2.9E-7 (8.2E-7)	3.0E-8 (1.2E-7)	1.4E-8 (4.1E-8)
Sb	4E-4	8.6E-4	8.6E-4	5.4E-7 (2.4E-6)	2.9E-7 (8.2E-7)	3.3E-7 (1.3E-6)	1.6E-7 (4.5E-7)	1.1E-8 (4.4E-8)	5.2E-9 (1.5E-8)
I	1E-2	3.0E-2	1.0E-2	8.7E-5 (3.9E-4)	4.6E-5 (1.3E-4)	1.1E-3 (4.6E-3)	5.4E-4 (1.5E-3)	6.1E-5 (2.5E-4)	2.9E-5 (8.3E-5)
Ba	2E-1	3.0E-2	2.9E-2	2.4E-5 (1.1E-4)	1.3E-5 (3.6E-5)	3.0E-5 (1.2E-4)	1.4E-5 (4.0E-5)	5.8E-4 (2.4E-3)	2.8E-4 (7.9E-4)
La	5E-5			1.6E-5 (**7.2E-5**)	8.4E-6 (2.4E-5)	3.1E-6 (1.3E-5)	1.5E-6 (4.2E-6)	3.3E-7 (1.3E-6)	1.6E-7 (4.5E-7)
W	8E-4			1.7E-6 (7.8E-6)	9.2E-7 (2.6E-6)	6.3E-7 (2.6E-6)	3.0E-7 (8.6E-7)	9.6E-8 (3.9E-7)	4.6E-8 (1.3E-7)
Hg	3E-4	5.7E-4	5.7E-4	6.0E-6 (2.7E-5)	3.2E-6 (9.1E-6)	1.1E-5 (4.3E-5)	5.0E-6 (1.4E-5)	6.9E-7 (2.8E-6)	3.3E-7 (9.3E-7)
Tl	1E-5		2.0E-5	1.0E-7 (4.5E-7)	5.3E-8 (1.5E-7)	3.7E-8 (1.5E-7)	1.8E-8 (5.1E-8)	9.9E-9 (4.0E-8)	4.7E-9 (1.3E-8)
Pb	2E-3	3.6E-3[b]		1.0E-5 (4.7E-5)	5.5E-6 (1.6E-5)	2.2E-5 (8.9E-5)	1.0E-5 (3.0E-5)	5.5E-6 (2.2E-5)	2.6E-6 (7.5E-6)
U	2E-4	8.6E-5	8.6E-5	4.2E-7 (1.9E-6)	2.2E-7 (6.4E-7)	8.7E-7 (3.5E-6)	4.1E-7 (1.2E-6)	1.4E-7 (5.6E-7)	6.5E-8 (1.9E-7)

[a] For inorganic As only. [b] Withdrawn.
